# Lack of Histamine H_4_-Receptor Expression Aggravates TNBS-Induced Acute Colitis Symptoms in Mice

**DOI:** 10.3389/fphar.2017.00642

**Published:** 2017-09-13

**Authors:** Eva J. Wunschel, Bastian Schirmer, Roland Seifert, Detlef Neumann

**Affiliations:** Institute of Pharmacology, Hannover Medical School Hanover, Germany

**Keywords:** TNBS-induced colitis, histamine, inflammation, receptor, GPCR, mouse models

## Abstract

Inflammatory bowel diseases (IBD) are a growing health problem worldwide, severely affecting patients’ life qualities and life expectancies. Therapeutic options, which are rare and focus on symptoms associated with the disease, suffer from increasing numbers of patients refractory to the established strategies. Thus, in order to generate new therapeutic regimens, the detailed understanding of the pathogenic mechanisms causing IBD is necessary. Histamine is an inflammatory mediator associated with IBD. Four histamine receptors are currently known of which the histamine H_4_-receptor (H_4_R) has been shown to possess a pro-inflammatory function in several experimental models of inflammatory diseases, including dextran sodium sulfate (DSS)-induced colitis in mice. No single model reflects the complexity of human IBD, but each model provides valuable information on specific aspects of IBD pathogenesis. While DSS-induced colitis mostly relies on innate immune mechanisms, trinitrobenzene sulfonic acid (TNBS)-induced colitis rather reflects T-cell mechanisms. Consequently, an observation made in a single model has to be verified in at least one other model. Therefore, in the present study we investigated the effect of genetic blockade of H_4_R-signaling in mice subjected to the model of TNBS-induced acute colitis. We analyzed severity and progression of clinical signs of colitis, as well as histopathologic alterations in the colon and local cytokine production. Genetic ablation of H_4_R expression worsened clinical signs of acute colitis and histological appearance of colon inflammation after TNBS application. Moreover, TNBS instillation enhanced local synthesis of inflammatory mediators associated with a neutrophilic response, i.e., CXCL1, CXCL2, and interleukin-6. Lastly, also myeloperoxidase concentration, indicative for the presence of neutrophils, was elevated in cola of TNBS-treated mice due to the absence of H_4_R expression. Our results indicate an anti-inflammatory role of histamine via H_4_R in TNBS-induced acute neutrophilic colitis in mice, thus questioning the strategy of pharmacological H_4_R blocked as new therapeutic option for patients suffering from IBD.

## Introduction

Inflammatory bowel diseases (IBD) are a global health concern with growing incidence, especially in industrialized countries (Molodecky et al., 2012). There are mainly two clinical entities of IBD, namely ulcerative colitis (UC) and Crohn’s disease (CD), both characterized by chronic, relapsing inflammatory conditions in the gastrointestinal tract. These two manifestations of IBD differ in their clinical and histopathological presentation and, from an immunological point of view, in the association of CD and UC with a dysregulation of a Th_1_-type or a Th_2_-type immune response, respectively (Geremia et al., 2014). But nowadays this dichotomic viewpoint needs to be reconsidered: Not only are there several published data indicating no clear Th_1_/Th_2_ phenotype in UC and CD (Vainer et al., 2000; Rovedatti et al., 2009; Bernardo et al., 2012). But during the last few years it has become evident that, beneath the adaptive Th_1_/Th_2_ immune response, constituents of Th_17_ response and mechanisms of innate immunity may play important roles in the pathophysiology of IBD, too (Geremia et al., 2014; Cătană et al., 2015).

There are several different rodent models existing in order to mimic specific features of human IBD. Because of their relative simplicity, easy establishment, low costs and good reproducibility, chemically induced colitis models are currently the most widely used models of IBD (Wirtz et al., 2007). When administered directly into the colon, the haptenizing agent 2,4,6-trinitrobenzene sulfonic acid (TNBS) elicits a Th_1_-polarized colonic immune response leading to wide-spread inflammation in the colon, which recapitulates several features of human IBD (Morris et al., 1989; Elson et al., 1996; Camoglio et al., 2000). Because of the Th_1_-polarization, this model has often been associated with human CD (Jones-Hall and Grisham, 2014). In dextran sodium sulfate (DSS)-induced colitis, the animals are fed with DSS in their drinking water, causing a direct toxic damage of the mucosal epithelial lining with loss of barrier integrity (Ni et al., 1996; Dharmani et al., 2011). Due to the morphological and symptomatic similarity, this model has been associated with human UC (Jones-Hall and Grisham, 2014). In contrast to DSS, TNBS does not directly damage the mucosal epithelium, but causes a delayed hypersensitivity reaction by haptenizing luminal antigens (Elson et al., 1996; Wirtz et al., 2007). Therefore, TNBS-induced colitis has been reported to focus on pathophysiologic aspects of the adaptive immune response, whereas DSS-induced colitis causes more of a dysregulation of the innate immune response (Wirtz et al., 2007).

One important inflammatory mediator involved in adaptive or innate immune responses is the biogenic amine histamine (2-(4-imidazolyl)-ethylamine), which can transduce its signal via four currently known histamine receptors H_1_R, H_2_R, H_3_R and H_4_R, all belonging to the superfamily of G protein-coupled receptors (GPCRs) (Seifert et al., 2013). The H_4_R is the most recently discovered histamine receptor and is expressed mainly on immune cells such as T-cells, eosinophils, mast cells and dendritic cells (Hofstra et al., 2003; Lundberg et al., 2011; Reher et al., 2012). Upon ligand binding, H_4_R couples to and activates G_i_ proteins leading to intracellular calcium mobilization and inhibition of membranous adenylyl cyclase activity (Morse et al., 2001; Leurs et al., 2009). The biological function of H_4_R remains elusive but accumulating evidence indicates a pro-inflammatory function of H_4_R in several (auto)inflammatory diseases like allergic asthma, atopic dermatitis and rheumatoid arthritis (Beermann et al., 2012; Cowden et al., 2014; Rossbach et al., 2016). Histamine has been shown to act as pro-inflammatory mediator in IBD (Bene et al., 2004; Neumann and Seifert, 2014) and blockade of H_4_R has proven to ameliorate DSS-induced colitis in mice as well as in TNBS-induced colitis in rats (Varga et al., 2005; Schirmer et al., 2015).

In a previous study, we detected a pro-inflammatory effect of H_4_R in DSS-induced acute colitis in BALB/cJ mice (Schirmer et al., 2015). Because DSS- and TNBS-induced colitis differ in their immunologic pathomechanisms, we hypothesized that the role of the H_4_R depends on the model used for induction of colitis. Therefore, in the present study the TNBS-induced acute colitis model was applied to H_4_R knockout and wild-type mice in order to elucidate a contextual function of the receptor.

## Materials and Methods

### Materials

If not stated otherwise, all chemicals were obtained from Sigma–Aldrich (Munich, Germany).

### Animals

Eight- to ten-week-old BALB/cJRj (WT) mice were purchased from Janvier Labs (Le Genest-Saint-Isle, France). H_4_R knockout mice (H_4_R^-/-^; strain: C.129HrH_4_^tm1Lex^) were generated by Lexicon Genetics (Woodlands, TX, United States) as described by Hofstra et al. (2003) and backcrossed for more than 10 generations onto the BALB/cJRj strain. Mice were housed in the animal facility of Hannover Medical School (temperature: 21°C ± 1; 14/10 h day/night cycle) with access to standard diet and drinking water *ad libitum*.

### Induction of Colitis by TNBS and Animal Dissection

For induction of acute colitis 8- to 10-week-old mice (male and female, weight: 20–25 g) were anesthetized (100 mg/kg ketamine plus 10 mg/kg xylazine; i.p.) and then inoculated intra-rectally (i.r.) with TNBS (2 mg/mouse) dissolved in 100 μl 45% [v/v] ethanol (EtOH) in PBS through a catheter inserted approx. 4 cm proximal to the anus. Control mice were treated with 100 μl EtOH/PBS only. Mice were inspected for their health status every 6 h. Three days after inoculation or when found with severely affected health status mice were killed with carbon dioxide and subsequent heart puncture. Caeca and cola were resected, washed with PBS to remove remaining feces, and the lengths of the cola were documented. Thereafter, the cola were transversally divided into three segments, proximal, medial, distal, and each segment was further longitudinally divided into 3 parts, the first comprising about ½ and parts two and three each about ¼ of the colonic circumference. The first part of each segment was fixed in 4% (v/v) formaldehyde (Merck, Darmstadt, Germany) embedded in paraffin, and processed for hematoxylin/eosin (H/E) staining. The second and a third part of each segment was stored at -80°C and in RNAlater (Thermo Fisher, Waltham, MA, United States), respectively, for protein and mRNA analyses.

### Evaluation of Disease Activity

Mice were examined at 24 h intervals and a disease activity index (DAI, adopted from Alex et al. (2009) ranging from 0 to 14 was employed based on total body weight loss (0: no weight loss, 1: ≤ 5%, 2: ≤ 10%, 3: ≤ 15%, 4: > 15%), stool consistency (0: normal, 2: soft, 4: diarrhea, 6: no defecation) and peranal bleeding (0: no bleeding, 2: little bleeding, 4: massive bleeding/no defecation).

### Histological Analysis

H/E-stained tissue slices were analyzed in a blinded fashion by two independent researchers. A histological severity score was calculated for each segment by evaluating the single parameters severity of inflammation (0: normal, 1: mild, 2: moderate, 3: severe, 4: highly severe), hyperplasia of crypts (0: absent, 2: present), degree of ulceration (0: no ulcers, 1: 1–2 ulcers involving up to 20 crypts, 2: 1–4 ulcers involving 20 to 40 crypts, 3: any ulceration exceeding the aforementioned criteria), area of affected tissue (0: 0%, 1: ≤ 30%, 2: ≤ 70%, 3: > 70%), edema formation (0: absent, 1: present), blood cell infiltration (0: absent, 1: present), and changes in crypt architecture (0: 0%, 1: ≤ 30%, 2: ≤ 70%, 3: > 70%). The total maximum score thus sums up to 17 per segment (Bleich et al., 2004). All *ex vivo* data reported in this manuscript refer to the medial colon segments since the tips of catheters used for TNBS administration reached this section after i.r. insertion. Thus, in accordance with the flow of the luminal content of the colon, in the medial and proximal colon segments substantial degrees of histopathological alterations due to TNBS application were found, while in the distal segments and in the caeca virtually no alterations could be observed.

### Cytokine Measurements

The frozen colon specimen were lysed at 4°C using a FastPrep24 device (MP Biomedicals, Santa Ana, CA, United States) and insoluble parts were removed by centrifugation (4°C, 10 min, 10,000*g*). Total protein concentrations of the cleared lysates were measured by BCA assay (Thermo Scientific). Concentration of interleukin (IL)-6, IL-17, CXCL1 (KC), CXCL2 (MIP-2), and tumor necrosis factor (TNF) was measured in the colon lysates using a customized multiplex magnetic Luminex Kit (R&D Systems, Minneapolis, MN, United States). In the same samples, myeloperoxidase (MPO) was quantified using a specific ELISA (R&D Systems).

### mRNA Quantification

The tissue specimen dedicated for mRNA analyses were lysed using the FastPrep24 device (MP Biomedicals) and the RNAs were extracted using the Nucleospin RNA II kit (Macherey-Nagel, Düren, Germany) essentially according to the manufacturer’s instructions. One microgram RNA of each sample were reverse transcribed for 30 min at 50°C into cDNA by means of Maxima Reverse Transcriptase (Thermo Scientific). Cytokine-specific sequences were quantified proportionately to GAPDH by PCR using the customized Primer PCR Assay (Bio-Rad, Munich, Germany).

### Statistical Analysis

Data are represented as single values and/or arithmetic mean of replicates ± SD for each parameter. Statistical analyses were performed with GraphPad Prism 6.07 using tests as indicated in the figure legends.

### Ethical Considerations

Animal housing and experimental procedures complied with the German Animal Welfare Legislation and were approved by the Lower Saxony State Office for Consumer Protection and Food Safety (LAVES, AZ 33.14-42502-04-14/1670).

## Results

**FIGURE 1:**
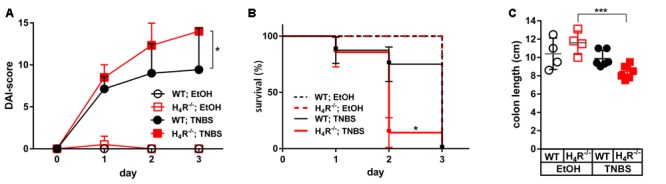
Lack of H_4_R expression worsens TNBS-induced colitis. Wild type (WT) or H_4_R-deficient (H_4_R^-/-^) BALB/cJ mice were treated intra-rectally with 2 mg/100 μl^∗^mouse TNBS (TNBS) or with an equivalent volume of the solvent mixture EtOH/PBS (EtOH) at day 0. Mice were inspected for their health status every 6 h for a total of 3 days. **(A)** Every 24 h the disease activity of each single mouse was evaluated according to a scoring system which takes into account the body weight, the stool consistency, and the degree of anal bleeding and assigns a numeric value to the respective status (Alex et al., 2009). The sum of the single values of a mouse refers to as its disease activity index (DAI)-score, being the higher the worse is the health condition of a mouse. ^∗^pWT; TNBS vs. H_4_R^-/-^; TNBS < 0.05 (Two-Way ANOVA), *n* = 7/group. **(B)** Mice which had to be euthanized due to severely impacted health status throughout the 3 days observation period were recorded. On day 3 all remaining mice were killed for analysis. The relative numbers of surviving mice were plotted against the time. ^∗^pWT; TNBS vs. H_4_R^-/-^; TNBS < 0.05 (Log-rank (Mantle-Cox) test), *n* = 7/group. **(C)** The cola of the mice were prepared and their lengths recorded. ^∗∗∗^<0.005 (One-Way ANOVA), TNBS: *n* = 7/group; EtOH: *n* = 4/group.

The application of 2 mg/mouse TNBS in WT mice resulted in a fast onset of cumulative clinical colitis symptoms, reported as DAI. Already 1 day after inoculation of TNBS, the DAI reached a value which did not significantly increase at day two and three. Colitis symptoms were virtually absent during the whole observation period in WT mice treated with the solvent EtOH/PBS (**Figure 1A**). The symptoms induced by TNBS were clearly dose-dependent, since the application of 1 mg/mouse TNBS lead to significantly reduced severity of symptoms (data not shown). In H_4_R^-/-^ mice, the symptoms induced by 2 mg/mouse TNBS were significantly more severe as compared to those observed in WT mice. Moreover, the DAI progressively increased in H_4_R^-/-^ mice from day one until day 3, while the EtOH/PBS-treated H_4_R^-/-^ mice remained without symptoms (**Figure 1A**). These data indicate that the absence of H_4_R expression promotes TNBS-induced colitis and were supported by the analysis of the survival rates of treated mice. All mice, WT and H_4_R^-/-^, treated with EtOH/PBS survived the observation period. Some of the TNBS-treated mice, however, had to be euthanized due to severely impaired health conditions within the 3 days period. Of those, the number of H_4_R^-/-^ mice (6/7) was significantly higher than that of WT mice (1/7) (**Figure 1B**). Lastly, also anatomical parameters support the notion that lack of H_4_R supports TNBS-induced colitis: while in WT mice experimentally induced colitis does not lead to a reduction of the colon length, in H_4_R^-/-^ mice it is significantly reduced after TNBS treatment as compared to EtOH/PBS treatment (**Figure 1C**).

**FIGURE 2:**
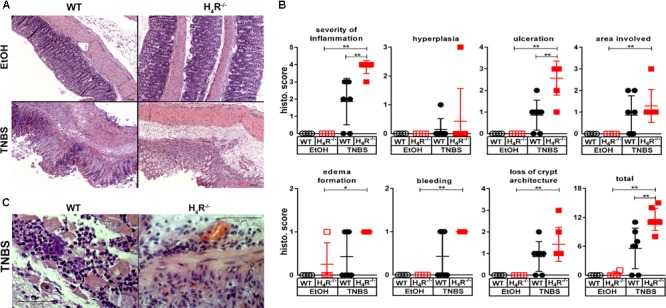
Lack of H_4_R expression enhances histopathological findings of colonic inflammation. Wild type (WT) or H_4_R-deficient (H_4_R^-/-^) BALB/cJ mice were treated with 2 mg/100 μl^∗^mouse TNBS (TNBS) or with an equivalent volume of the solvent mixture EtOH/PBS (EtOH). After dissection, the cola were cut in three sections, proximal, medial, and distal. Parts of the sections were fixed in formaldehyde, embedded in paraffin, cut into slices, and stained with hematoxylin/eosin. **(A)** Representative micro-photographs of tissue slices of medial colon sections of each experimental group are demonstrated. **(B)** The tissue slices as demonstrated in **(A)** were analyzed in a blinded fashion for pathological derangements as indicated above the single graphs applying a scoring matrix, i.e., the higher the score, the worse the histological appearance. ^∗^*p* < 0.05, ^∗∗^*p* < 0.01, ^∗∗∗^*p* < 0.005 (One-Way ANOVA), TNBS: *n* = 7/group; EtOH: *n* = 4/group. **(C)** Representative micro-photographs in enhanced magnification of sections obtained from cola of TNBS-treated mice.

The histopathological analysis of the cola at the end of the observation period revealed an elevated degree of derangements in TNBS-treated WT mice as compared to their counterparts treated with the solvent EtOH/PBS (**Figure 2A**). The absence of H_4_R expression did not alter the histopathological appearance in solvent-treated mice, while TNBS-treated H_4_R^-/-^ mice were significantly stronger affected than the respective WT mice as detected by quantitative evaluation of the specimen (**Figure 2B**). In detail, TNBS treatment, as compared to instillation of the solvent, induced a significant increase in all parameters analyzed except hyperplasia of crypts in H_4_R^-/-^ mice, while in WT mice these values were increased as well but without statistical significance (**Figure 2B**). The comparison of TNBS-treated WT and H_4_R^-/-^ mice revealed significant differences for the parameters severity of inflammation and degree of ulceration (**Figure 2B**). Two out of the seven mice in the TNBS-treated WT group did not show histopathological derangements. Excluding these two from the calculations as outliers, nevertheless, did not alter the statistical significance of the differences between WT and H_4_R^-/-^ mice (data not shown). TNBS-induced acute colitis is a local inflammatory reaction with a predominant involvement of neutrophils (Campbell et al., 2016). Neutrophils represent the major cell type found in the inflammatory infiltrations in cola of TNBS-treated WT and H_4_R^-/-^ mice (**Figure 2C**). A direct quantification of the neutrophilic infiltrates in order to compare WT and H_4_R^-/-^ mice was not performed, but substituted by the more accurate surrogate parameter MPO concentration, which represents a quantitative measure for not only the number of infiltrated neutrophils but also their activation (see below).

**FIGURE 3:**
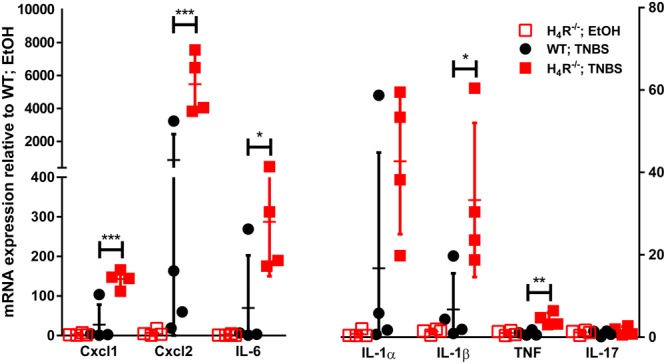
Lack of H_4_R expression enhances mRNA expression of inflammation-associated cytokines and chemokines. Wild type (WT) or H_4_R-deficient (H_4_R^-/-^) BALB/cJ mice were treated with 2 mg/100 μl^∗^mouse TNBS (TNBS) or with an equivalent volume of the solvent mixture EtOH/PBS (EtOH). After dissection, the cola were cut in three sections, proximal, medial, and distal. From parts of the medial sections mRNAs were extracted and analyzed by RT-qPCR array. Relative abundancies of individual mRNAs as indicated on the abscissa were calculated relative to the abundancy of the GAPDH mRNA. Reported are the values comparative to those observed in solvent-treated WT mice, which were set to 1. ^∗^*p* < 0.05, ^∗∗^*p* < 0.01, ^∗∗∗^*p* < 0.005 (Student’s *t*-test), *n* = 4/group.

**FIGURE 4:**
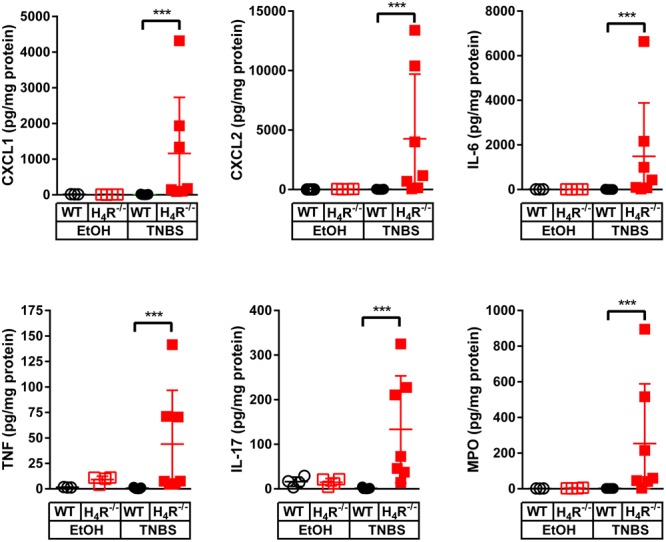
Lack of H_4_R expression enhances protein expression of inflammation-associated cytokines and chemokines. Wild type (WT) or H_4_R-deficient (H_4_R^-/-^) BALB/cJ mice were treated with 2 mg/100 μl^∗^mouse TNBS (TNBS) or with an equivalent volume of the solvent mixture EtOH/PBS (EtOH). After dissection, the cola were cut in three sections, proximal, medial, and distal. From parts of the medial sections proteins were extracted and analyzed by Luminex Array (cytokines, chemokines) and ELISA (MPO). Analyte concentrations are reported relative to the total protein concentrations of the colon lysates. ^∗∗∗^*p* < 0.005 (One-Way ANOVA), TNBS: *n* = 7/group; EtOH: *n* = 4/group.

In order to more closely characterize the colitis reactions occurring in WT and H_4_R^-/-^ mice, the expression of inflammatory mediators, esp. those attracting and activating neutrophils, was analyzed in colon tissues. In analyses on the mRNA level a tendency to TNBS-induced expression of CXCL1, CXCL2, IL-6, IL-1α, and IL-1β was observed in WT mice, while the expression of other genes such as TNF and IL-17 was only marginally altered (**Figure 3**). Of those mediators induced by TNBS, the mRNA expression of CXCL1, CXCL2, and IL-6 as well as of IL-1β and TNF were affected by the absence of the H_4_R, i.e., the expression was enhanced in cola of TNBS-treated H_4_R^-/-^ mice as compared to cola obtained from WT mice (**Figure 3**). On the protein level, similar observations were made. The concentrations of CXCL1, CXCL2, and IL-6 were significantly enhanced in tissues obtained from TNBS-inoculated H_4_R^-/-^ mice as compared to that of WT mice (**Figure 4**). Moreover, also the pro-inflammatory mediators TNF and IL-17 and the neutrophil-specific enzyme MPO, indicative for a neutrophilic inflammation, were detected at significantly higher protein concentrations after TNBS treatment in cola of H_4_R^-/-^ mice than in cola of WT mice.

Thus, not only clinical symptoms but also histological and immunological parameters indicate that rectally applied TNBS induces a much more severe neutrophilic colitis in H_4_R^-/-^ mice as compared to WT mice.

## Discussion

In several models of inflammatory diseases blockade of H_4_R signaling provides a beneficial effect on inflammatory symptoms (Dunford et al., 2006; Cowden et al., 2010a,b, 2014; Gutzmer et al., 2011; Hartwig et al., 2015; Kovacova-Hanuskova et al., 2015). Accordingly, also in models of inflammation of the gut, H_4_R was reported to possess a pro-inflammatory function (Varga et al., 2005; Schirmer et al., 2015). However, these two studies were performed in two different models. The first one used TNBS to induce colitis in rats and analyzed the involvement of H_4_R by application of the selective antagonist JNJ7777120 (Varga et al., 2005). The other study was performed using the model of DSS-induced colitis in mice taking advantage of a genetic H_4_R knockout model (Schirmer et al., 2015). Thus, a series of parameters (species, colitis induction method, H_4_R blockade method) differed profoundly between these two studies, disabling the extrapolation of the results of the one study to the other. Moreover, no single model reflects the complexity of human IBD, but each model provides valuable information on specific aspects of IBD pathogenesis. Thus, only data obtained in several models will provide evidence whether or not a proposed strategy may be useful in treating human IBD. This prompted us to analyze the involvement of H_4_R in acute TNBS-induced colitis in mice using the genetic knockout (H_4_R^-/-^) model.

Surprisingly, both clinical and histopathological examination of the TNBS-treated mice demonstrated not only the progressive occurrence of symptoms indicative for experimental colitis in WT mice, but, in comparison to these, also a severe aggravation in mice lacking H_4_R expression. These data were supported by the reduced mean survival time, a pathological parameter valid for this model but not for human IBD, and the reduced colon length of TNBS-treated H_4_R^-/-^ mice as compared to solvent-treated mice. Thus, in the TNBS-induced model, colonic inflammation seems to be suppressed by H_4_R signaling, as already described for the model of experimental autoimmune encephalomyelitis (EAE) (Ballerini et al., 2013; Saligrama et al., 2013).

In contrast to the study by Varga et al. (2005) we analyzed the contribution of H_4_R to TNBS-induced acute colitis in mice, but not in rats, and we inhibited H_4_R function by genetic ablation of H_4_R expression, but not by application of a H_4_R-selective antagonist. Since the immune systems of mice and rats, including the mechanisms of inflammation, work pretty much the same, the different species rather cannot account for the contrary H_4_R functions in the two models. Specific microbiota and age of an animal both affect experimental diseases, including colitis (Hansen et al., 2014). However, these parameters, which cannot be compared between the two studies, only gradually regulate the disease and, thus, also very unlikely may account for the controversial differences observed. Regarding the method of H_4_R blockade, in response to the genetic ablation of the receptor compensatory or other secondary mechanisms may have been evoked, which do not occur upon application of a pharmacological antagonist such as JNJ7777120. However, at least for a series of immunologic parameters, naïve WT and H_4_R^-/-^ mice do not differ significantly from each other (Hartwig et al., 2015; Kloth and Neumann, unpublished results). In contrast, for the pharmacological activity of the H_4_R-selective antagonist JNJ7777120 mouse strain-related differences have already been described in a model of acute skin inflammation (Coruzzi et al., 2012), which also may account for the differences between the mice- and the rat-based studies.

An even more straightforward comparison can be drawn between the present study and that using the DSS-induced acute colitis model (Schirmer et al., 2015), which both took advantage of the same colony of WT and H_4_R^-/-^ BALB/cJ mice. Thus, these two studies only differ specifically in the method of acute colitis induction, i.e., by intrarectal TNBS application (this study) and by DSS feeding (Schirmer et al., 2015), indicating that the opposite findings regarding the function of H_4_R can be assigned to the different methods. In both models, which were driven over a rather short period of time, i.e., 3 days (TNBS) and 7 days (DSS), the observed symptoms and parameters are primarily based on the induction of intestinal epithelial damage and acute inflammation. Due to the protocol used, the adaptive immune system cannot have become activated and therefore only plays a minor role in these models, while in other models it is undeniably important (van Lierop et al., 2010). This indicates that the applied DSS and TNBS methods activate different mechanisms, which both result in colitis symptoms. In support of this hypothesis, differing profiles of cytokine expression were already detected in DSS- and TNBS-induced acute colitis (Alex et al., 2009). The bases for the differences of H_4_R function in DSS- and TNBS-induced colitis in mice, however, have still to be explored.

In the present study, the chemokines CXCL1 (KC) and CXCL2 (MIP2) together with the cytokine IL-6 and the neutrophils enzyme MPO were abundantly expressed in the cola after TNBS application, and their expression was enhanced in mice lacking H_4_R expression. In addition, in sera obtained from TNBS-treated H_4_R^-/-^ mice higher levels of CXCL1, CXCL2, and IL-6 were detected as compared to those from WT mice, albeit these differences were statistically not significant (Supplementary Figure S1). Whether this is a direct or an indirect effect of H_4_R on the mediator-releasing cells has to be analyzed in the future. Nevertheless, the regulation of the expression of the neutrophils attracting chemokines CXCL1, CXCL2, and of IL-6 and MPO connects H_4_R to the neutrophilic inflammatory reaction, and thereby to innate immune mechanisms. Cellular sources for CXCL1, CXCL2, and IL-6 are monocytes and macrophages, implying that these cells express a functional H_4_R. While functional expression of H_4_R in macrophages has already been demonstrated by us and others (Cowden et al., 2013; Czerner et al., 2014), H_4_R expression on monocytes currently is controversially discussed (Gschwandtner et al., 2013; Dib et al., 2014; Werner et al., 2014; Capelo et al., 2016). Nevertheless, at least in macrophages H_4_R function is described as pro-inflammatory, thus, probably cannot account for the anti-inflammatory effect in TNBS-induced colitis. Another colonic cellular source that releases CXCL1, CXCL2, and IL-6 upon activation is epithelial cells (Song et al., 1999; Ohtsuka et al., 2001; Sutton et al., 2008). Thus, one can hypothesize that histamine via the H_4_R regulates the expression of neutrophils attracting/activating mediators by epithelial cells and thereby affects the inflammatory reaction. Unfortunately, H_4_R expression and function on colonic epithelial cells remain to be analyzed in detail yet.

Certainly, one major drawback of the present study is the rather low number of animals in the experimental groups. These were calculated to be sufficient when planning the described experiments in advance; however, the obtained data indicate that an enhanced number would have been needed. Unfortunately, due to legislation hurdles it is hardly possible to get a permission to just enhance the number of animals of an already existing study, limiting the strength of the data’s interpretation.

In summary, in the present study we demonstrate that lack of H_4_R expression worsens the outcome of TNBS-induced acute colitis in mice, indicating an anti-inflammatory function of H_4_R in this model. The cellular and molecular bases for this function are still enigmatic; however, our data question the general view on H_4_R-selective antagonists as potential new anti-inflammatory therapeutics.

## Author Contributions

Substantial contributions to the conception or design of the work: BS and DN. Substantial contributions to the conception or design of the work; or the acquisition, analysis: EW, BS, and DN. Substantial contributions to the conception or design of the work; or the acquisition, analysis, or interpretation of data for the work: EW, BS, RS, and DN. The work of drafting: BS and DN. The work of drafting it or revising it critically for important intellectual content: EW, BS, RS and DN. Final approval of the version to be published: EW, BS, RS, and DN. Agreement to be accountable for all aspects of the work in ensuring that questions related to the accuracy or integrity of any part of the work are appropriately investigated and resolved: EW, BS, RS, and DN.

## Conflict of Interest Statement

The authors declare that the research was conducted in the absence of any commercial or financial relationships that could be construed as a potential conflict of interest.
