# Supplementary material for: Lack of Histamine H4-Receptor Expression Aggravates TNBS-Induced Acute Colitis Symptoms in Mice
Source: Front Pharmacol. 2017 Sep 13;8:642. doi: 10.3389/fphar.2017.00642 (PMC5601386; doi:10.3389/fphar.2017.00642)
Supplement: Supplementary file 1 [file Presentation_1.PDF]

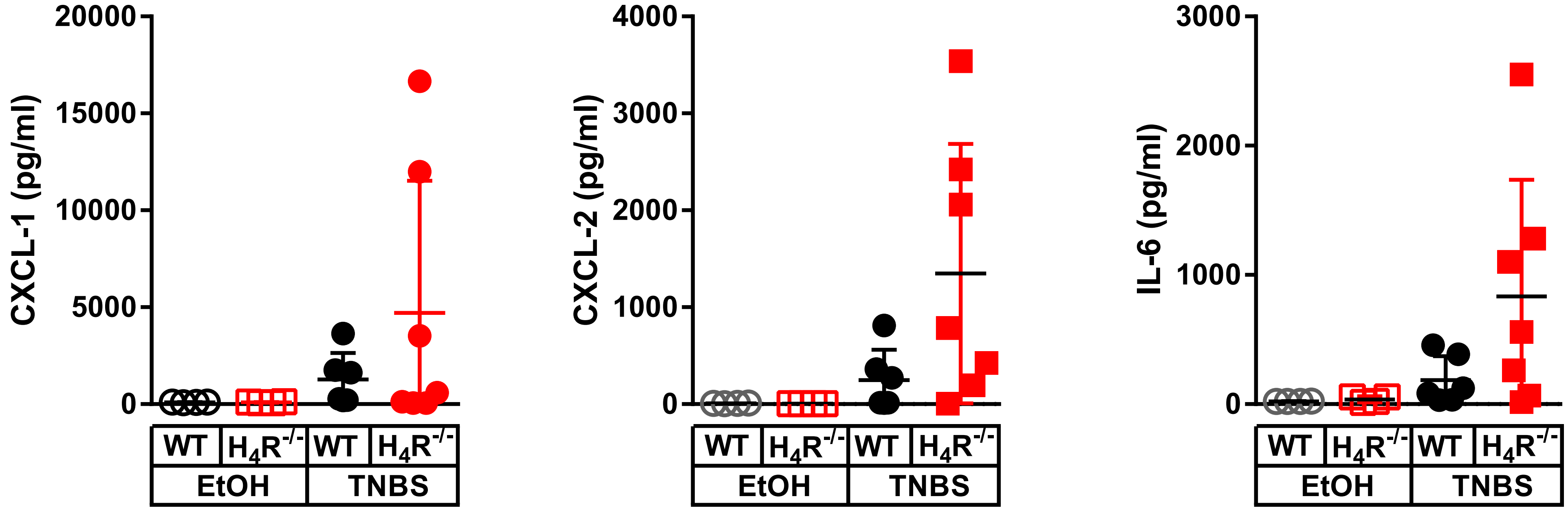

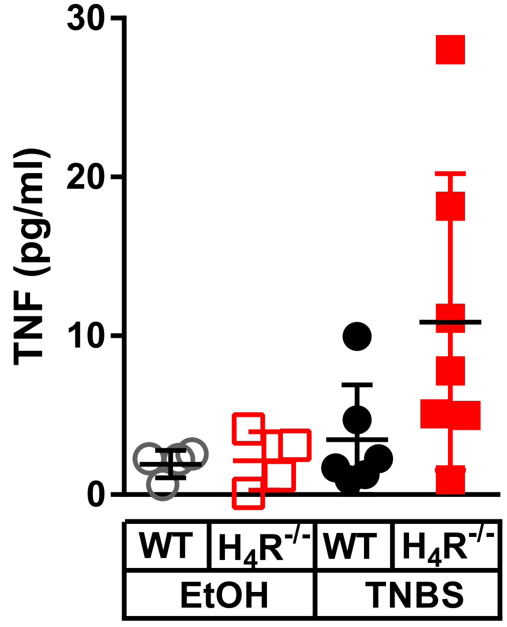

Figure S1. Lack of H_4R expression enhances synthesis of inflammation-associated cytokines and chemokines. Wild type (WT) or H_4R-deficient ($H_4R^{-/-}$) BALB/cJ mice were treated with 2mg/100µl*mouse TNBS (TNBS) or with an equivalent volume of the solvent mixture EtOH/PBS (EtOH). Sera were prepared and cytokine concentrations were analyzed by Luminex Array. Differences were all without statistical significance.
